# Workplace inequality is associated with status-signaling expenditure

**DOI:** 10.1073/pnas.2115196119

**Published:** 2022-04-08

**Authors:** Naomi Muggleton, Anna Trendl, Lukasz Walasek, David Leake, John Gathergood, Neil Stewart

**Affiliations:** ^a^Department of Social Policy and Intervention, University of Oxford, Oxford OX1 2ER, United Kingdom;; ^b^Brasenose College, University of Oxford, Oxford OX1 4AJ, United Kingdom;; ^c^Warwick Business School, University of Warwick, Coventry CV4 7AL, United Kingdom;; ^d^Department of Psychology, University of Warwick, Coventry CV4 7AL, United Kingdom;; ^e^School of Economics, University of Nottingham, Nottingham NG7 2RD, United Kingdom

**Keywords:** income inequality, status signaling, social rank, digital footprints

## Abstract

Regional inequality is known to magnify sensitivity to social rank. This, in turn, is shown to increase people’s propensity to acquire luxury goods as a means to elevate their perceived social status. Yet existing research has focused on broad, aggregated datasets, and little is known about how individual-level measures of income interact with inequality within peer groups to affect status signaling. Using detailed financial transaction data, we construct 32,008 workplace peer groups and explore the longitudinal spending and salary data associated with 683,677 individuals. These data reveal links between people’s status spending, their absolute salary, salary rank within their workplace peer group, and the inequality of their workplace salary distribution. Status-signaling luxury spending is found to be greatest among those who have higher salaries, whose workplaces exhibit higher inequality, and who occupy a lower rank position within the workplace. We propose that low-rank individuals in unequal workplaces suffer status anxiety and, if they can afford it, spend to signal higher status.

## Significance

Scholars have identified that inequality is a notable detriment to well-being. Status-signaling luxury expenditure is taken as a symptom of the reduced well-being associated with income inequality. Despite evidence that status-signaling luxury expenditure is higher in unequal regions, it remains unclear who is affected by inequality. We use payroll and daily spending data from 683,677 individuals in 32,008 precisely-defined workplace peer groups to show that workers at unequal firms spend significantly more on high-status, luxury goods. This is also seen in those with a high absolute salary, but low salary rank within their workplace. Compared to aggregated, regional data, financial data allow us to identify groups of workplace peers and offer precise measurements of status-signaling expenditure for each individual.

Economic inequality has grown substantially in recent years across the world, with 70% of the global population experiencing rising levels of income disparity (1). As well as concerns surrounding meritocracy and equality of opportunity (2–4), there is growing interest in the potential impact that rising inequalities might have on health, societal, and economic outcomes. It is now well established that inequality is associated with increased political polarization (5), higher rates of obesity and diabetes (6), weaker educational performance (7), and lower life expectancy (8), among many other negative outcomes (9–12). One prominent explanation for these findings is the status anxiety hypothesis, which posits that, in the presence of high income inequality, people feel more threatened about, and pay more attention to, their position in the social hierarchy (11, 13). The psychological stress that accompanies the need to monitor and improve one’s social status fosters narcissism, a sense of entitlement, and expression of self-enhancement values (10, 14, 15). At the same time, high levels of inequality elevate the role of money in expressing one’s worth, which motivates people to engage in conspicuous consumption and the purchasing of positional goods. In other words, the status anxiety hypothesis predicts that, when inequality is high, people devote more resources (both economic and psychological) to the pursuit of goods that can function as signals of one’s wealth and income, such as luxury brands and expensive possessions. Indeed, expenditure on luxurious, high-status items is higher in unequal regions, suggesting that conspicuous goods may be regarded as salient markers of success (13, 16–18). These effects are also consistent with the findings that individuals living in more-unequal regions borrow more and save less, in part to finance their desire to keep up with the Joneses (19–21).

The intuitive appeal of this perspective is apparent: Income inequalities elevate concerns with one’s income-defined status, which is reflected in a preoccupation with luxury goods. Yet many critical questions remain unanswered, as the existing research does not yet specify the conditions under which inequality can influence one’s consumption. Much of the prior work on status consumption has relied on aggregate behavior across thousands or millions of individuals to identify correlations between inequality and some measure of the interest or pursuit of positional goods (22–24). But aggregating over large geographical regions prevents one from controlling for individuals’ absolute income and income rank. This is problematic for two reasons. First, it relies on ecologically fallacious reasoning. For example, it is possible that spending on luxury goods in unequal regions may be driven merely by those who can afford it (i.e., those with the highest incomes). It is therefore possible that the effects of inequality on the pursuit of luxury could be explained by the higher number of rich individuals in unequal regions. To understand whether the aggregate-level evidence for the status anxiety hypothesis applies to the individual, individual-level data must be used. The second issue stems from the empirical finding that satisfaction with one’s income is best predicted by income rank, not the absolute amount one earns (25). Consequently, it remains unclear whether the rise of conspicuous consumption in response to inequality is driven by those who occupy lower- or higher-rank positions in the income distribution. Without understanding how income inequality relates to the level and rank of income, our understanding of inequality and status anxiety is incomplete.

Here we uncover the complex relationship between individual-level inequality and spending behavior. We achieve this by leveraging our unique access to mass-transactional banking data from a large UK retail bank. We combine two key data assets: 1) the accurate tracking of luxury expenditure across individuals for 10 mo using mass transactional spending data; 2) the precise measurement of inequality and income rank (specifically, salary) among these individuals, who constitute small peer groups of, on average, 28 coworkers in a firm, via payroll data. To classify luxury expenditure, we draw upon electronic transactions associated with 4,118 merchants according to their merchant category and subcategory descriptions (*SI Appendix*, Table S1). All transactions were classified as luxuries, discretionaries, necessities, or unknown. Luxury merchant categories include hotels, airlines, antiques, jewelry, champagne retailers, and furriers. Eleven percent of transactions could not be classified and were excluded from further analysis. Exemplar merchants are given in Table 1 (for definitions, see *SI Appendix*, Tables S1–S3).

**Table 1.** Exemplar merchants by expenditure group: 30 merchants that are illustrative of luxury, discretionary, and necessity expenditure

Expenditure	Description
Luxury	
British Airways	Airline
Center Parcs	Tourism
Booking.com	Hotel
Gett	Taxi
Land Rover	Motor
Marriott	Hotel
Pandora	Jewelry
Sky TV	TV subscription
Sotheby’s	Art and antiques
Uber	Taxi
Discretionary	
Apple App Store	Entertainment
Costa	Coffee shop
Debenhams	Department store
Google Play	Entertainment
JD Wetherspoons	Pub
John Lewis	Department store
Just Eat	Food delivery
Pret a Manger	Sandwich shop
Starbucks	Coffee shop
Very	Clothing
Necessity	
Asda	Supermarket
Boots	Pharmacy
British Gas	Utilities
Direct Line	Car insurance
Lidl	Supermarket
Shell	Petrol
Superdrug	Pharmacy
Transport for London	Commuter
TV License	Utilities
Vision Express	Opticians

**Fig. 1.**
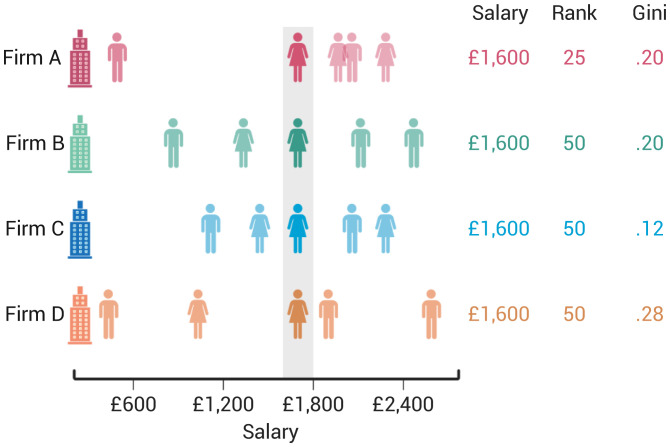
Computation of rank and inequality. Individuals belong to a peer group (firm *j*), which comprises peers who receive different salaries (*x* axis). Based on their position in the peer group’s salary distribution, each individual in firm *j* is assigned a rank between 0 (lowest salary in peer group) and 100 (highest salary in peer group). Based on the dispersion of salaries within a peer group, all individuals within firm *j* are assigned the same inequality value, a value between 0 (perfect equality) and 1 (perfect inequality). All four target individuals (highlighted in gray) receive the same salary (£1,600), but differ in their peer group inequality and comparative rank within their peer group.

Fig. 1 is an illustration of how inequality (here, Gini coefficient) and rank are calculated at the monthly level in our data. This example shows four target individuals (see gray shading) from four different peer groups. Each target individual has a salary of £1,600 per month but differs in her salary rank (pink: 25th percentile; green, blue, and orange: 50th percentile) and peer group inequality (blue: low; pink and green: medium; orange: high). Higher inequality indicates a more unequal distribution of salaries (compare firm C, low inequality, and firm D, high inequality, in Fig. 1).

We consider who purchases luxury goods when inequality is high. To do this, we test how luxury spending is predicted by absolute salary, salary rank position within the firm, and firm inequality.

## Results

**Fig. 2.**
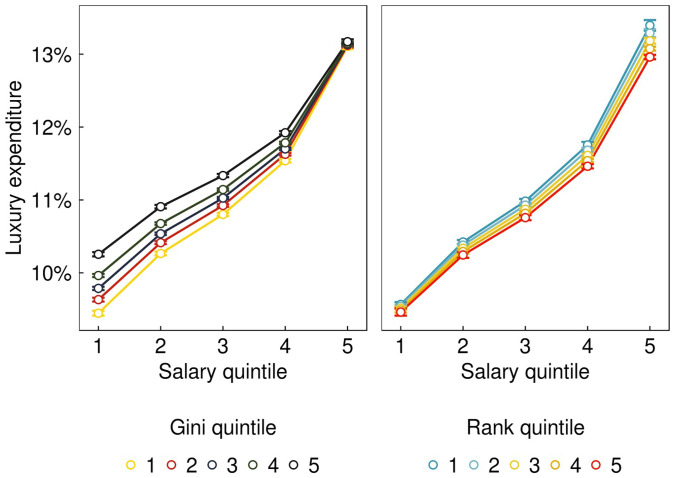
Fitted proportion of spending on luxury goods by salary and Gini (*Left*, from model 1) and salary and rank salary (*Right*, from model 2). Spending is on purchases at *t* + 1 across 683,677 individuals, between March and December 2019. Individuals are binned by their net salary in month *t* and their peer group inequality measured by Gini (*Left*) or peer group rank salary (*Right*). Salary, Gini, and rank bins were determined by cutting each variable into five equally sized quintile bins. Higher Gini quintiles (in black) denote individuals from firms with highly unequal salaries. Higher rank quintiles (in red) denote individuals with the highest salaries within their firm. Error bars are 95% CIs.

We modeled the interaction between salary, rank, and inequality, plus demographic, controls using the individual-level data. The sample comprised 683,677 individuals in 32,008 workplaces, across 10 mo of spending. Table 2 reports two models, estimating the relationship between luxury spending (as a proportion of total expenditure) and workplace inequality (model 1) and the rank position of the individual’s salary within the firm (model 2). SEs are robust, addressing the possible nonindependence of months within an individual. Model 1 shows that luxury expenditure is positively associated with salary and workplace inequality measured by Gini. These main effects are qualified by a salary-by-Gini interaction: Fig. 2, *Left* shows that the effect of Gini is smaller at higher salaries.

**Table 2.** Linear regression (*n* = 683,677) of proportion of expenditure spent on luxury goods, as a function of 1) workplace Gini and the Gini × Salary interaction and 2) one’s salary rank within the workplace and the Rank × Salary interaction

	1			2		
Variable	*B*		*SE*	*B*		*SE*
Intercept	0.10060	***	0.00041	0.10007	***	0.00041
Salary	0.01437	***	0.00013	0.01465	***	0.00017
Gini	0.00211	***	0.00012			
Salary × Gini	–0.00146	***	0.00011			
Rank				–0.00086	***	0.00015
Salary × Rank				–0.00049	***	0.00012
Gender (woman = 0)	0.02372	***	0.00024	0.02319	***	0.00024
Age	0.00003	**	0.00001	0.00005	***	0.00001
*R* ^2^			0.01225			0.01207

Both models control for an individual’s salary, age, and gender. SEs in the regressions are robust, clustered by individual. *B*, standardized regression coefficient; ***P < 0.001; **P < 0.01.

Model 2 also shows that luxury expenditure is positively associated with salary, with the coefficient almost unchanged from model 1. There is a main effect of rank salary, such that the proportion of spending on luxury goods reduces for those with higher ranking salary. These main effects are qualified by a salary-by-rank interaction. The negative coefficients on rank and the salary-by-rank interaction mean that the effect of salary rank is larger for those with higher salaries (Fig. 2, *Right*).

To test the role of gender as a moderating factor, we reran all analysis separately for men and women. This showed that these effects of inequality and rank position on luxury spending are moderated by gender. Specifically, the luxury expenditure of men is sensitive to inequality within the firm, whereas the luxury expenditure of women is sensitive to rank position within the firm (*SI Appendix*, Table S4). This is an insight into gender differences in status-signaling behavior. However, we did not have a theoretical reason to anticipate this effect, so we report for robustness separate analyses for each gender (*SI Appendix* and Fig. S1).

As a robustness check, we replicated these findings with industry sector and subsector fixed effects, to control for a scenario where, say, a management consultants’ luxury expenditure is artificially high, owing to a reliance on hotels, taxis, and airplanes for his or her work (*Robustness Checks*).

## Discussion

This paper used objective transaction-level data from 683,677 individuals to reveal the association between inequality and status-seeking expenditure. Our results reveal complex relationships between luxury expenditure and one’s salary, salary rank, and inequality. Status-signaling luxury spending is found to be greatest among those who have higher salaries, whose workplaces exhibit higher inequality, and who occupy a lower rank position within the workplace.

Taken together, our results advance our understanding of how inequality promotes status-seeking behavior at the level of the individual. By using granular transactional data, we can identify who in the salary distribution is particularly sensitive to rank and inequality, thereby providing a richer understanding of how, and who within, society is particularly sensitive to inequality. We see that high earners are more sensitive to rank position, but low earners are more sensitive to inequality. This supports prior work, which posits that luxury spending is a signal by which individuals can improve their status and, consequently, their self-esteem and self-worth (16–19). We also observe that individuals with low peer group status but higher salaries spend comparatively more on luxury goods. This observation is in line with the predictions formed by the status anxiety hypothesis, which anticipates that status anxiety (here, low rank) combined with the means to purchase more expensive goods (measured here as high salary) will result in a higher proportion spent on luxury goods.

The present findings demonstrate the relationship between inequality and rank in precisely defined peer groups. Previous work has typically relied on observational data to approximate peer groups at the level of cities, states, or countries. By identifying peer groups of, on average, 28 individuals per group, we show the association between one’s immediate reference group and individual spending behavior. Further, by using person-level, rather than aggregate, data, we identified which members of the peer group are driving the increased consumption of status-seeking goods. The prior work which has relied on group-level measures shows that regional inequality increases consumption of, and online searches for, luxury brands and goods (22, 23, 26). Yet aggregate data do not allow researchers to identify who in the peer group is driving increased status competition.

The present study has a number of limitations that could be addressed with further study. First, our sample is composed of individuals who were in work for each of the 10 mo in our observation period (1 March to 31 December 2019). As such, the results might not generalize to individuals who are working intermittently, or who work for multiple employers and thus have multiple, concurrent peer groups. Second, although our sampling restrictions attempted to capture individuals for whom we have a comprehensive view of spending behavior, we do not rule out the possibility that individuals have alternative means for paying for their essential and nonessential expenditures. Third, we are unable to measure the well-being implications of our findings. For example, high-ranking individuals spend higher sums on luxury goods when placed in an unequal peer group. We do not, however, determine whether this behavior is associated with higher or lower subjective well-being. Individuals may experience utility from their elevated status (27, 28). Alternatively, these individuals might be averse to inequality and experience negative emotions such as guilt. Given the objective, observational nature of our data, although measuring spending with great accuracy, we do not reliably infer subjective well-being. Similarly, we cannot measure bank customers’ levels of anxiety, nor whether this is the sole factor shaping individuals’ higher luxury expenditure. Future research may be able to combine survey data with transactional data to shed light on these relationships (notwithstanding the challenges of obtaining large samples of survey data with matched administrative data). Such data might allow tests of whether luxury expenditure is symptomatic of status-signaling behavior or a generalized preference for expensive items.

The findings also raise questions about the role of small peer groups in shaping the social, health, and economic outcomes that have been documented in the status anxiety literature. Prior work has explained the relationship between political polarization (5), obesity and diabetes (6), weaker educational performance (7), and lower life expectancy (8), among other negative outcomes (9–12), through the status anxiety hypothesis. The association between peer group and status-enhancing expenditure that we observe here suggests that future research exploring the relationship between one’s peer group rank and broader outcomes could be fruitful.

## Conclusion

We find that status-seeking expenditure is positively associated with peer group inequality. This relationship is robust and particularly strong among individuals with a low rank among peers. These results raise the possibility that status-seeking spending is a marker of rank insecurity in peer groups where inequality and rank are salient. Exploring the impact of these findings will have important implications for our understanding of how inequality affects subjective well-being, societal hierarchy, and the role of consumer debt in society.

## Materials and Methods

### Ethical Approval.

The Privacy Risk and Impact Assessment Committee at the retail bank granted ethical approval for the study. Upon opening an account, all customers consented for their data to be used for research. The Humanities and Social Sciences Research Ethics Committee at the University of Warwick waived the requirement for an additional ethics review, as in cases where appropriate ethical review has already taken place at another collaborating institution, so as to avoid unnecessary duplication.

### Expenditure Data.

Spending behavior is measured by electronic transactions to merchants identified by the bank in its typology of transactions. A transaction is defined as any spending behavior that occurs using a debit card or credit card. This includes electronic transfers, online transactions, and chip and pin or contactless in-store transactions, but neither cash transactions nor checks.

Each spending transaction is associated with a merchant string denoting the name of the seller, of which there were 4,118 in our sample. These merchants are categorized into one of four categories: necessity, discretionary, luxury, and unknown. Of all of the transactions occurring during our observation window, 11% were classified as unknown. Of the remaining transactions, 24% were tagged as necessity, 54% as discretionary, and 10% as luxury. Merchants are classified according to their merchant category and subcategory descriptions (terms are provided in *SI Appendix*, Tables S1–S3). This was constructed independently of the authors and prior to the analysis commencing. The preexisting classification that is reported here is the only classification that the authors analyzed.

To construct a measure of spending, we classified all spending transactions conducted by a customer with a merchant. This excludes interaccount and intraaccount transfers, as well as payments to friends and family. Spending behavior was observed between 1 April 2019 and 31 December 2019; 1 April 2019 represents the first date that the bank began to utilize the classification system that identified spending as being luxury, discretionary, or necessity spending. December 2019 represents the last full calendar month prior to data analysis beginning. Transaction amounts by spending type tag (necessity, discretionary, luxury, or unknown) were aggregated at the monthly level and divided by the total monthly spending. For example, if individual *i* in month *t* spent £500 at necessity, £400 at discretionary, £100 at luxury, and £50 at unclassified merchants, then their total spending = £1,000, and the proportion spent is defined as necessity (£500/£1,000) = 0.50, discretionary (£400/£1,000) = 0.40, and luxury (£100/£1,000) = 0.10. Unclassified spending was excluded from this calculation (i.e., removed from the denominator) to avoid a scenario where individuals with high volumes of unclassified spending have artificially low values for necessity, discretionary, and luxury spending. As such, the denominator represents the sum of necessity, discretionary, and luxury spending for individual *i* in month *t*, which means that the proportion of luxury, discretionary, and necessity spending sums to one.

Our primary dependent variable is the luxury expenditure, because the main prediction of the status anxiety hypothesis is a positive association between inequality and spending on positional goods. But, since our data contain records of all transactions, we report results for discretionary and necessity expenditure as well. *SI Appendix*, Table S5 shows that the median proportion spent on luxury goods and services was 0.03 (mean = 0.12). For discretionary expenditure, the median was 0.41 (mean = 0.42). For necessity expenditure, the median was 0.46 (mean = 0.46).

### Payroll Data.

Payroll data are measured by electronic transactions from firms identified by the bank in its typology of transactions. Payroll names are aggregated, such that subtle variations in company name are merged. For example, should a company change its name from “ABC Ltd.” in month *t* to “ABC and Co. Ltd.” in month *t* + 1, the firms are grouped as “ABC” across the observation period. This resulted in the inclusion of 66,965 firms across 11 sectors and 56 subsectors.

Salary was calculated as the total inflows (after tax) from firm *j* to individual *i* in month *t*. If *i*’s payment cycle was weekly, all payments made from firm *j* in month *t* were aggregated to give a value for monthly salary. The upper and lower 1% of salary (£6,849 and £201) was removed. Rank refers to an individual’s position on the salary ladder at firm *j* in month *t*. Inequality was defined as the Gini coefficient across all salaries at firm *j* in month *t*. Summary statistics for payroll are presented in *SI Appendix*, Table S6. To aid the interpretation of regression coefficients, salary, rank, and inequality are standardized such that the mean = 0 and SD = 1.

### Sample Selection.

#### Inclusion criteria.

Our analyses contain a representative sample of the in-work UK population. Of the 52.4 million adults in the United Kingdom, 1.5 million (2.9%) are unbanked. Our in-scope sample was ∼10.6% of the adult UK population. We used the retail bank’s definition of an active customer as an individual whose account(s) process at least 12 transactions per month. This definition was constructed independently of the authors and prior to the analysis commencing. Internal work at the retail bank has shown that 12 is the optimal minimum threshold for estimating whether a customer is active or inactive. The definition avoids including cases where individuals hold dormant bank accounts. The inclusion criteria also ensured that all individuals were aged 18 y or older during the observation time frame. This was to avoid potential ethical implications of conducting research on underage persons.

#### Exclusion criteria.

Our sample consisted of a sample of the in-work population of the United Kingdom. To avoid small-sample biases of the Gini coefficient (29), we first identified all UK-based firms (*j*) with 10 or more employees who banked with the retail bank in at least one given month between 1 March 2019 and 1 December 2019 (*N_j_* = 83,502). These dates reflect one lagged month prior to the expenditure data. This accounts for the fact that we lagged our independent variables so that salary, rank, and inequality at time *t* are used to predict spending at *t* + 1. Individuals who worked in an “unidentified” sector were excluded, as this is often indicative of payment portals used to pay contractors, such as umbrella companies. As such, these individuals often do not have contact with other individuals with whom they share payroll data, and so are not peers. After this step, our sample included 72,168 firms.

Next, for all firms, we identified any individuals (*i*) who received a regular income from firm *j* (*N_i_* = 6,205,787). Participants with payroll data missing for some or all months were removed. This removed individuals who were unemployed, retired, on unpaid sick leave, or on maternity/paternity leave for one or more months, but did not remove individuals who changed firms during the observation period. For those individuals who changed firms, we redefined their peer group in the month that their employment changed. After this step, our sample included 4,296,954 individuals and 66,667 peer groups.

Finally, to ensure that peers were working in close proximity (e.g., in the same office), we sought to exclude those who didn’t work for small to medium firms, defined as 250 employees (30). Based on the bank’s market share, we inferred that this equated to 100. As such, we excluded individuals who worked in firms with more than 100 coworkers in a given month. This excludes employees for large firms with multiple sites across the United Kingdom. Our final sample comprised 683,677 individuals from 32,008 peer groups. The mean peer group size was 28.29 (median = 21).

### Model Specifications.

The dependent variable is the proportion of an individual’s monthly spending that is classified as luxury spending. The independent variables were salary, rank, and inequality, plus all interaction terms, and the individual’s age and gender as control variables. The unit of analysis in this sample was an individual calendar month. We lagged our independent variables so that salary, rank, and inequality at time *t* are used to predict spending at *t* + 1.

Model 1 takes the form

[1] $${\text{luxury}}_{it+1}=\beta_{S}X_{S(it)}+\beta_{G}X_{G(it)}+\beta_{SG}X_{S(it)}X_{G(it)}+X_{C}\beta_{C(i)}+\epsilon_{it+1},$$

And model 2 takes the form

[2] $${\text{luxury}}_{it+1}=\beta_{S}X_{S(it)}+\beta_{R}X_{R(it)}+\beta_{SR}X_{S(it)}X_{R(it)}+X_{C}\beta_{C(i)}+\epsilon_{it+1},$$

where ${\text{luxury}}_{it+1}$ is the dependent variable indicating the proportion of individual *i*’s monthly spending that was tagged as being a luxury, *S* refers to the salary term, *G* refers to the Gini coefficient term, and *R* refers to the salary rank term, while $X_{C}$ is the matrix of covariates, including age and gender. *β* is the coefficient for a given term, while $\epsilon_{it+1}$ is the error term. The equations for discretionary and necessity spending are identical to Eqs. **1** and **2**, with the only change being that of switching the dependent variables to ${\text{discretionary}}_{it+1}$ and ${\text{necessity}}_{it+1}$, respectively.

### Robustness Checks.

#### Necessity and discretionary spending.

If our findings are consistent, we should expect to find opposing effects for necessity spending relative to luxury spending. We should also find that the effects for discretionary spending lie somewhere between the effects observed for luxury and necessity spending. To test whether our results were consistent, we replicated our findings in *SI Appendix*, Tables S7 and S8.

#### Occupation effects.

As a robustness check, we replicated these findings with industry sector and subsector fixed effects (*SI Appendix*, Tables S9 and S10) to control for a scenario where, say, a management consultants’ luxury expenditure is artificially high, owing to a reliance on hotels, taxis, and airplanes for his or her work. Additionally, we replicated the findings by excluding workers from the subsector “investments,” which contains occupations such as asset or wealth management, investment banking, and hedge fund management (*SI Appendix*, Table S11).

#### Definitions of necessity spending.

Purchases at some necessity-labeled vendors could reflect necessities or luxuries (e.g., bananas vs. champagne at the supermarket). To control for potentially luxurious expenditure at merchants labeled as providing necessities, we conducted sensitivity analyses with either supermarket, hospital, dental, or motor spending excluded from one’s total spending (*SI Appendix*, Tables S12–S15).

## Supplementary Material

Supplementary File

## Data Availability

Data cannot be shared. The bank data cannot be available in an openly accessible data repository. For peer review purposes, we can provide access to the data for the purpose of running the code to replicate our results. This can be achieved via virtual private network access to the local server on which the data are stored (and on which the analysis is run) under supervision of one of the authors of the paper. We can also provide local access to an independent researcher on site at the universities of Warwick or Oxford, United Kingdom.
