# Supplementary material for: Workplace inequality is associated with status-signaling expenditure
Source: Proc Natl Acad Sci U S A. 2022 Apr 8;119(15):e2115196119. doi: 10.1073/pnas.2115196119 (PMC9169648; doi:10.1073/pnas.2115196119)
Supplement: Supplementary File [file pnas.2115196119.sapp.pdf]

Supplementary Information for

Workplace inequality is associated with status-signalling expenditure

Naomi Muggleton, Anna Trendl, Lukasz Walasek, David Leake, John Gathergood, and Neil Stewart

Corresponding Author: Naomi Muggleton.
E-mail: naomi.muggleton@spi.ox.ac.uk

This PDF file includes:

Supplementary text
Fig. S1
Tables S1 to S15

Supporting Information Text

We have found that the effect of inequality and salary rank within a firm interact with gender. This finding was not the focus of our analysis and we did not have a theoretical motivation for expecting these gender differences. Instead, the possibility of gender differences in salary led us to check the robustness of our results for men and women separately, and in doing so we reveal a pattern where men, but not women, are sensitive to inequality and women, but not men, are sensitive to rank. The first two columns of S4 show a large main effect of Gini (inequality) for men but a much smaller effect for women. The last two columns show a large main effect of salary rank for women but a much smaller effect for men. S1 visualises these effects. The main effect of Gini for women but not men is evident in the spacing, or lack of spacing, between the lines in the left panels. The Gini-by-salary interaction is such that the effect of Gini is smaller at higher salaries. The main effect of rank for men but not women is evident in the spacing, or lack of spacing, between the lines in the right panels. The rank-by-salary interaction is such that the effect of rank is larger at higher salaries. These serendipitous findings for gender are intriguing and require theoretical explanation, but should be taken with caution: Gender is correlated with many economic variables, and there will be differences by gender and salary in who selects into having an active sole (rather than joint) account, and thus who selects into appearing in our dataset.

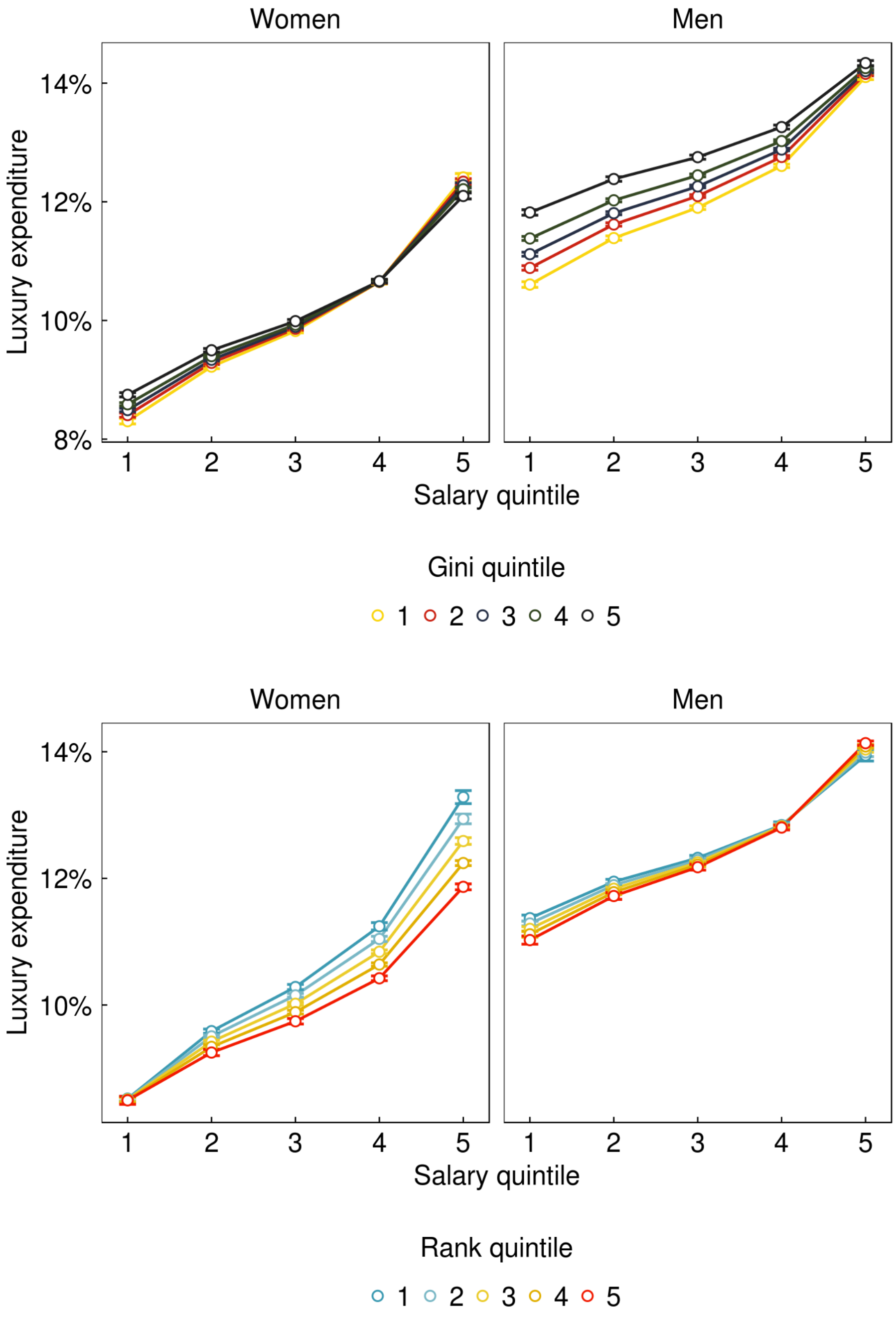

Fig. S1. Fitted proportion of spend on luxury goods by salary and Gini (left, from Model 1) and salary and salary rank (right, from Model 2), separated for women and men. Spend is on purchases at $t + 1$ across 683,677 individuals, between March – December 2019. Individuals are binned by their net salary in month t and their peer group inequality measured by Gini (left) or peer group salary rank (right). Salary, Gini, and rank bins were determined by cutting each variable into five equally-sized quintile bins. Higher Gini quintiles (in black) denote individuals from firms with highly unequal salary. Higher rank quintiles (in red) denote individuals with the highest salaries within their firm. Error bars are 95% CIs

Table S1. Luxury expenditure. Definition of luxury purchases.

Category	Subcategory
Airline	All
Hotels & accommodation	All
Vehicle dealers / servicing	All
Travel	All except: ‘passenger railway’ and ‘local commuter transport’
Clothing stores	Furriers & fur shops
DIY stores	Lumber / build supply stores
DIY stores	Hardware equipment / supply
Other	Telecommunication equipment
Other	Cable / pay TV services
Other	Marinas, service & supply
Other retail stores	Champagne stores
Other retail stores	Antique reproduction stores
Other retail stores	Swimming pools / sales / servicing
Other retail stores	Jewellery stores
Other retail stores	Art dealers and galleries

Table S2. Discretionary expenditure. Definition of discretionary purchases.

Category	Subcategory
Household stores	All
Pubs, restaurants, recreation	All
Clothing stores	All except: 'furriers & fur shops'
Food stores	pkg stores / beer / wine / liquor
Food stores	Candy / nut confection store
DIY stores	All except: 'lumber / building supply stores' and 'hardware equipment / supplies'
Other retail stores	All except: 'chemicals / allied prods', 'commercial equipment', 'commercial furniture', 'construction materials', 'dental / lab / med equipment', 'industrial supplies', 'office / photo equipment', 'inbound telemarketing merchant', 'outbound telemarketing merchant', 'drug stores & pharmacies', 'orthopaedic goods', 'pet stores / food & supply', 'hearing aids / sales / service', 'champagne stores', 'antique reproduction stores', 'art dealers & galleries', 'swimming pools / sales / servicing', 'jewellery stores'
Services	Beauty / barber shops
Services	Dry cleaners

Table S3. Necessity expenditure. Definition of necessity purchases.

Category	Subcategory
Travel	Passenger railway
Travel	Local commuter transport
Petrol stations	All
Food stores	All except: ‘pkg stores / beer / wine / liquor’ and ‘candy / nut confection store’
Financial services	Insurance sales / underwrite
Financial services	Direct marketing insurance
Other	Telecommunication services
Other	Utilities / electricity / gas / water / sanitary
Other retail stores	Drug stores & pharmacies
Other retail stores	Orthopaedic goods
Other retail stores	Pet stores / food and supply
Other retail stores	Hearing aid / sales / service
Services	Child care services
Services	Funeral services / crematories
Services	Hospitals
Services	Heating, plumbing, air conditioning
Services	Electrical contractors
Services	Dentists / orthodontists
Services	Doctors / physicians
Services	Medical / dental labs
Services	Medical / health services
Services	Opticians

Table S4. Linear regression of proportion of expenditure spent on luxury goods, as a function of (1) workplace Gini and the Gini × Salary interaction, and (2) one's salary rank within the workplace, and the Rank × Salary interaction. Both models control for an individual's salary, and age, and are modelled separately for men and women.

	Luxury spend							
	(1)				(2)			
	Women		Men		Women		Men	
	β	SE	β	SE	β	SE	β	SE
Intercept	$.11967^{***}$	.00054	$.11070^{***}$	.00055	$.11971^{***}$	.00054	$.10882^{***}$	.00055
Salary	$.01629^{***}$	.00020	$.01315^{***}$	.00017	$.01777^{***}$	.00026	$.01238^{***}$	.00023
Gini	$.00044^{***}$	.00017	$.00345^{***}$	.00017				
Rank					$-.00245^{***}$	.00022	$-.00036^{**}$	.00021
Age	$-.00042^{***}$	.00001	$.00036^{***}$	.00001	$-.00039^{***}$	.00001	$.00038^{***}$	.00001
Salary × Gini	$-.00149^{***}$	.00017	$-.00193^{***}$	.00015				
Salary × Rank					$-.00224^{***}$	.00018	$.00087^{***}$	.00017
R^2	.00799		.00592		.00810		.00556	

β, standardised regression coefficient. Probability values: $^{***}p < .001$; $^{**}p < .01$; $^{*}p < .05$.

Table S5. Summary statistics for expenditure data. Summary statistics for spending data by purchase type for all individual $\times$ months. Panel A reports monthly expenditure in pounds. Panel B reports monthly expenditure as a proportion of total monthly expenditure. SD denotes standard deviation. N states total number of individuals in the sample.

			Percentiles				
	Mean	SD	*p*25	*p*50	*p*75	*p*90	*p*99
Panel A: Monthly expenditure (£)							
Luxury	178.88	819.46	0.00	22.31	116.74	361.99	2454.591
Discretionary	406.48	542.98	118.59	273.22	517.41	873.10	2321.361
Necessity	399.71	399.06	150.18	313.03	546.84	834.33	1601.920
Panel B: Monthly expenditure (%)							
Luxury	.12	.18	.00	.03	.14	.35	.86
Discretionary	.42	.24	.25	.41	.58	.74	1.00
Necessity	.46	.24	.28	.46	.64	.80	1.00
N = 683,677							

Table S6. Summary statistics for payroll data. Salary is calculated per individual $\times$ month (N = 6,275,509); N colleagues and Inequality are calculated per firm $\times$ month (N = 274000). SD denotes standard deviation. N states total number of individuals in the sample.

			Percentiles				
	Mean	SD	*p*25	*p*50	*p*75	*p*90	*p*99
Salary (£)	1,918.98	3,815.93	1,195.33	1,613.28	2,241.83	3,103.92	6,849.05
Inequality (Gini)	.24	.09	.18	.23	.29	.36	.55
N colleagues*	28.29	19.64	14	21	35	58	93
N = 683,677							

*N colleagues who bank with the retail bank. Note that, due to data limitations, in some cases we do not see the full size of a given firm.

Table S7. Linear regression of proportion of expenditure spent on discretionary goods, as a function of (1) workplace Gini and the Gini × Salary interaction, and (2) one's salary rank within the workplace, and the Rank × Salary interaction. Both models control for an individual's salary, age, and gender.

	Discretionary spend			
	(1)		(2)	
	β	SE	β	SE
Intercept	$.60458^{***}$	$.00063$	$.60045^{***}$	$.00064$
Salary	$.01527^{***}$	$.00019$	$.01534^{***}$	$.00025$
Gini	$.01203^{***}$	$.00018$		
Rank			$-.00476^{***}$	$.00022$
Gender (woman = 0)	$-.03345^{***}$	$.00039$	$-.03596^{***}$	$.00039$
Age	$-.00399^{***}$	$.00001$	$-.00393^{***}$	$.00001$
Salary × Gini	$-.00205^{***}$	$.00015$		
Salary × Rank			$.00475^{***}$	$.00017$
R^2	$.05984$		$.05808$	

β, standardised regression coefficient. Probability values: $^{***}p < .001$; $^{**}p < .01$; $^{*}p < .05$.

Table S8. Linear regression of proportion of expenditure spent on necessity goods, as a function of (1) workplace Gini and the Gini × Salary interaction, and (2) one's salary rank within the workplace, and the Rank × Salary interaction. Both models control for an individual's salary, age, and gender.

	Necessity spend			
	(1)		(2)	
	β	SE	β	SE
Intercept	$.29483^{***}$	.00070	$.29948^{***}$	.00070
Salary	$-.02964^{***}$	.00020	$-.02999^{***}$	.00027
Gini	$-.01414^{***}$	.00020		
Rank			$.00563^{***}$	.00025
Gender (woman = 0)	$.00973^{***}$	.00043	$.01277^{***}$	.00043
Age	$.00396^{***}$	.00002	$.00388^{***}$	.00002
Salary × Gini	$.00351^{***}$	.00017		
Salary × Rank			$-.00426^{***}$	.00019
R^2	.05932		.05671	

β, standardised regression coefficient. Probability values: $^{***}p < .001$; $^{**}p < .01$; $^{*}p < .05$.

Table S9. Linear regression of proportion of expenditure spent on luxury goods, as a function of (1) workplace Gini and the Gini × Salary interaction, and (2) one's salary rank within the workplace, and the Rank × Salary interaction. Both models control for an individual's salary, age, and gender, and contain sector fixed effects.

	Luxury spend			
	(1)		(2)	
	β	SE	β	SE
Salary	$.01451^{***}$	$.00013$	$.01484^{***}$	$.00018$
Gini	$.00225^{***}$	$.00012$		
Rank			$-.00096^{***}$	$.00015$
Gender (woman = 0)	$.02354^{***}$	$.00025$	$.02314^{***}$	$.00025$
Age	$.00003^{***}$	$.00001$	$.00006^{***}$	$.00001$
Salary × Gini	$-.00152^{***}$	$.00011$		
Salary × Rank			$-.00050^{***}$	$.00012$
R^2	$.01237$		$.01217$	

β, standardised regression coefficient. Probability values: $^{***}p < .001$; $^{**}p < .01$; $^{*}p < .05$.

Table S10. Linear regression of proportion of expenditure spent on luxury goods, as a function of (1) workplace Gini and the Gini × Salary interaction, and (2) one's salary rank within the workplace, and the Rank × Salary interaction. Both models control for an individual's salary, age, and gender, and contain subsector fixed effects.

	Luxury spend			
	(1)		(2)	
	β	SE	β	SE
Salary	$.01401^{***}$	.00013	$.01397^{***}$	.00018
Gini	$.00213^{***}$	.00012		
Rank			$-.00038^{***}$	.00015
Gender (woman = 0)	$.02316^{***}$	.00025	$.02289^{***}$	.00025
Age	$.00005^{***}$	.00001	$.00008^{***}$	.00001
Salary × Gini	$-.00144^{***}$	.00011		
Salary × Rank			$-.00036^{***}$	.00012
R^2	.01296		.01278	

β, standardised regression coefficient. Probability values: $^{***}p < .001$; $^{**}p < .01$; $^{*}p < .05$.

Table S11. Linear regression of proportion of expenditure spent on luxury goods, as a function of (1) workplace Gini and the Gini × Salary interaction, and (2) one's salary rank within the workplace, and the Rank × Salary interaction. Both models control for an individual's salary, age, and gender. Model excludes employees working in the investment subsector.

	Luxury spend			
	(1)		(2)	
	β	SE	β	SE
Intercept	.10049***	.00041	.09994***	.00041
Salary	.01437***	.00013	.01461***	.00017
Gini	.00207***	.00012		
Rank			−.00084***	.00015
Gender (woman = 0)	.02374***	.00024	.02322***	.00024
Age	.00003***	.00001	.00005***	.00001
Salary × Gini	−.00150***	.00011		
Salary × Rank			−.00045***	.00012
R^2	.01223		.01205	

β, standardised regression coefficient. Probability values: $^{***}p < .001$; $^{**}p < .01$; $^{*}p < .05$.

Table S12. Linear regression of proportion of expenditure spent on luxury goods after excluding supermarkets from total spend, as a function of (1) workplace Gini and the Gini × Salary interaction, and (2) one's salary rank within the workplace, and the Rank × Salary interaction. Both models control for an individual's salary, age, and gender.

	Luxury spend			
	(1)		(2)	
	β	SE	β	SE
Intercept	$.11219^{***}$	$.00047$	$.11221^{***}$	$.00047$
Salary	$.01389^{***}$	$.00015$	$.01369^{***}$	$.00020$
Gini	$.00042^{***}$	$.00014$		
Rank			$.00036^{**}$	$.00017$
Gender (woman = 0)	$.02951^{***}$	$.00028$	$.02939^{***}$	$.00028$
Age	$.00052^{***}$	$.00001$	$.00053^{***}$	$.00001$
Salary × Gini	$-.00134^{***}$	$.00012$		
Salary × Rank			$-.00096^{***}$	$.00013$
R^2	$.01200$		$.01198$	

β, standardised regression coefficient. Probability values: $^{***}p < .001$; $^{**}p < .01$; $^{*}p < .05$.

Table S13. Linear regression of proportion of expenditure spent on luxury goods after excluding hospitals from total spend, as a function of (1) workplace Gini and the Gini × Salary interaction, and (2) one's salary rank within the workplace, and the Rank × Salary interaction. Both models control for an individual's salary, age, and gender.

	Luxury spend			
	(1)		(2)	
	β	SE	β	SE
Intercept	$.09938^{***}$	.00041	$.09885^{***}$	.00041
Salary	$.01436^{***}$	.00013	$.01458^{***}$	.00017
Gini	.00209	.00012		
Rank			$-.00079^{***}$	.00015
Gender (woman = 0)	$.02378^{***}$	.00024	$.02327^{***}$	.00024
Age	$.00005^{***}$	.00001	$.00007^{***}$	.00001
Salary × Gini	$-.00146^{***}$	.00011		
Salary × Rank			$-.00045^{***}$	.00012
R^2	.01236		.01217	

β, standardised regression coefficient. Probability values: $^{***}p < .001$; $^{**}p < .01$; $^{*}p < .05$.

Table S14. Linear regression of proportion of expenditure spent on luxury goods after excluding dental-related transactions from total spend, as a function of (1) workplace Gini and the Gini × Salary interaction, and (2) one's salary rank within the workplace, and the Rank × Salary interaction. Both models control for an individual's salary, age, and gender.

	Luxury spend			
	(1)		(2)	
	β	SE	β	SE
Intercept	$.09925^{***}$	.00041	$.09872^{***}$	.00041
Salary	$.01444^{***}$	.00013	$.01468^{***}$	.00017
Gini	$.00212^{***}$	.00012		
Rank			$-.00082^{***}$	.00015
Gender (woman = 0)	$.02387^{***}$	.00024	$.02334^{***}$	.00024
Age	$.00006^{***}$	.00001	$.00009^{***}$	.00001
Salary × Gini	$-.00147^{***}$	.00011		
Salary × Rank			$-.00046^{***}$	.00012
R^2	.01242		.01223	

β, standardised regression coefficient. Probability values: $^{***}p < .001$; $^{**}p < .01$; $^{*}p < .05$.

Table S15. Linear regression of proportion of expenditure spent on luxury goods after excluding cars from total spend, as a function of (1) workplace Gini and the Gini × Salary interaction, and (2) one's salary rank within the workplace, and the Rank × Salary interaction. Both models control for an individual's salary, age, and gender.

	Luxury spend			
	(1)		(2)	
	β	SE	β	SE
Intercept	$.09687^{***}$	.00039	$.09614^{***}$	.00039
Salary	$.01322^{***}$	.00012	$.01418^{***}$	.00017
Gini	$.00274^{***}$	.00011		
Rank			$-.00194^{***}$	.00014
Gender (woman = 0)	$.01133^{***}$	.00023	$.01061^{***}$	.00023
Age	$-.00023^{***}$	.00001	$-.00020^{***}$	.00001
Salary × Gini	$-.00104^{***}$	.00010		
Salary × Rank			$-.00024^{**}$	.00011
R^2	.00879		.00857	

β, standardised regression coefficient. Probability values: $^{***}p < .001$; $^{**}p < .01$; $^{*}p < .05$.
